# Examination of a Nervous Patient

**Published:** 1912-05

**Authors:** Hansell Crenshaw

**Affiliations:** Atlanta, Ga.; Neurologist to the Hospital for Nervous Diseases, Atlanta; Neurologist to the Wesley Memorial Hospital; Professor of Physiology in the Southern College of Pharmacy


### EXAMINATION OF THE NERVOUS PATIENT.

By Hansell Crenshaw, M. D., Atlanta, Ga.

*Neurologist to the Hospital for Nervous Diseases, Atlanta; Neurologist to the Wesley Memorial Hospital; Professor of Physiology in the Southern College of Pharmacy.*

Probaby the chief reason why diagnoses of nervous disorders are so often incorrect is that the examinations of nervous patients are seldom systematic and complete. In no other field of medicine is a broad, thorough and orderly study of each case so imperative as in neurology. This is true in a measure because in no other field do we encounter such clever simulation of symptoms. Moreover, nervous manifestations, more than any other, owe their origin to disorders of other kinds, past or present.

The diagnosis will almost make itself if the examination be sufficiently comprehensive and systematic. In mapping out a plan for the study of cases, four major courses of inquiry must be provided for: namely: Family history, patient's history, malady's history, and present condition of the patient. Nothing short of an exhaustive inquiry along each of these lines constitutes a thorough examination into the case. The order in which these several lines of inquiry is taken up is immaterial, some physicians preferring to begin with the family history, while other commence with the physical examination of the patient. But some particular order should be chosen and adhered to. Personally, I begin by asking for the chief complaint. I follow this by going into the history of the present disorder, with special reference to onset and duration. Next, I investigate the personal history of the patient, beginning with intra-uterine life, if possible. This investigation includes inquiry concerning infancy, childhood, illnesses, injuries, habits, occupation, shocks, matrimonial status, and age. I then take up the family history, and inquire about the longevity of ancestors, the health of ancestors and relatives, and the diseases they have had. The list of diseases includes neuroses, psychoses, alcoholism, drug addictions, syphilis, tuberculosis, "apoplexy," and "rheumatism." Finally comes the physical examination of the present condition of the patient. This examination begins with the cranium and follows a downward regional sequence to the soles of the feet; and is supplemented by urinalysis and such examination of the blood as the circumstances seem to warrant. No organ escapes interrogation, but the most searching scrutiny is brought to bear upon "that outpost of the nervous system," the eye. Also, no superficial or deep reflex is overlooked.

In examining a neurotic individual we may do irreparable damage with our questions. The danger lurks in the likelihood of implanting in impressionable persons by suggestions the symptoms about which we inquire. In this way, persistent pains, anaesthesias, or even contractures may be induced by unwise interrogations. It is really never best to ask the patient directly concerning subjective symptoms. If there be pain, he will mention it. If there be anaesthesia, you will discover it when the patient fails to react the point of the pin. And if there be paralysis or paresis, the handclasp, reflexes, and so forth, will disclose it.

Furthermore, the less questions we ask, the fewer misleading answeres we invite. Relatives or intimate friends of the patient may be plied freely with questions, provided the patient is out of ear-shot.

A more productive method of investigation, however, is to start the patient on a complete account of his case and simply let him talk. Thus not only may we gain specific knowledge of his subjective symptoms, but also a very fair estimate of his mental and moral caliber.

In suitable cases, the patient may be directed to prepare a brief written account of his trouble. Here the character of the handwriting and mode of expression may prove illumining. Indeed, no examination is complete of a nervous individual without a recent specimen of his or her script, unless the patient is illiterate.

The more the careful physician has to do with the study of the nervous and insane, the less is his inclination toward snap-judgment and "snap-shot diagnosis." He knows that even with the utmost care, masters of diagnosis often fail; that several maladies often co-exist; and that the only salvation of the diagnostician lies in system and broad perspective.

1027 Candler Bldg.

